# Role of innate immunity-triggered pathways in the pathogenesis of Sickle Cell Disease: a meta-analysis of gene expression studies

**DOI:** 10.1038/srep17822

**Published:** 2015-12-09

**Authors:** Bidossessi Wilfried Hounkpe, Maiara Marx Luz Fiusa, Marina Pereira Colella, Loredana Nilkenes Gomes da Costa, Rafaela de Oliveira Benatti, Sara T Olalla Saad, Fernando Ferreira Costa, Magnun Nueldo Nunes dos Santos, Erich Vinicius De Paula

**Affiliations:** 1 Faculty of Medical Sciences, University of Campinas/Hematology and Hemotherapy Center, Campinas, SP, Brazil; 2 Department of Clinical Pathology, University of Campinas, Campinas, SP, Brazil

## Abstract

Despite the detailed characterization of the inflammatory and endothelial changes observed in Sickle Cell Disease (SCD), the hierarchical relationship between elements involved in the pathogenesis of this complex disease is yet to be described. Meta-analyses of gene expression studies from public repositories represent a novel strategy, capable to identify key mediators in complex diseases. We performed several meta-analyses of gene expression studies involving SCD, including studies with patient samples, as well as in-vitro models of the disease. Meta-analyses were performed with the Inmex bioinformatics tool, based on the RankProd package, using raw gene expression data. Functional gene set analysis was performed using more than 60 gene-set libraries. Our results demonstrate that the well-characterized association between innate immunity, hemostasis, angiogenesis and heme metabolism with SCD is also consistently observed at the transcriptomic level, across independent studies. The enrichment of genes and pathways associated with innate immunity and damage repair-associated pathways supports the model of erythroid danger-associated molecular patterns (DAMPs) as key mediators of the pathogenesis of SCD. Our study also generated a novel database of candidate genes, pathways and transcription factors not previously associated with the pathogenesis of SCD that warrant further investigation in models and patients of SCD.

Sickle cell disease (SCD) is a genetic disorder that affects approximately 300,000 newborns worldwide each year, mostly in developing countries^1^. Early diagnosis and improvements in supportive care allow more patients to survive into adulthood, thereby increasing the burden of this condition. It has been estimated that by 2050, the lives of nearly 10 million patients with SCD will be saved, leading to a major increase in the prevalence of this condition^2^. Since the majority of SCD patients reside in low and medium income countries, the provision of adequate care to SCD patients should be regarded as one of the most important healthcare challenges of the next decades. Despite significant improvements during the last decades, the disease remains associated with unacceptably high morbidity and mortality.

Although SCD is caused by a single amino acid substitution in the β chain of hemoglobin, the disease is characterized by multisystem and progressive organ damage affecting almost every system of the body^3^. Such widespread consequences are explained by the systemic and sustained inflammatory response observed in SCD, whose triggers and perpetuators are subject of intense investigation. In fact, despite the detailed characterization of several discrete parts of this inflammatory response, the hierarchical relationship between all these elements is yet to be described^4^,^5^.

High-throughput genomic technologies such as microarrays have contributed to our understanding of complex interactions in multisystem diseases such as diabetes and cancer^6^,^7^. In SCD, two microarray-based gene expression studies were published in the last year in different populations of patients^8^,^9^. In addition, this technology has also been used in the study of the effect of heme on endothelial cells (EC)^10^. Microarray-based studies generate large databases of raw gene expression data that are deposited in data repositories for public reuse^11^. Recently, meta-analysis of these data emerged as an attractive strategy to generate new biological insights that could not be obtained from individual studies^12^. In analogy to role of meta-analysis in the clinical arena, the combined analysis of gene expression datasets has the potential to reduce study biases and increase statistical power, obtaining a more accurate estimate of differentially expressed (DE) genes^12^,^13^. Moreover, the last years have witnessed the development of several new bioinformatics tools capable to generate more complex and biologically relevant data from lists of DE genes. These tools allow the prediction of biological pathways, protein-protein interactions, kinase and transcription factor regulatory networks, thus contributing to the generation of new hypothesis about the pathogenesis of complex traits^14^.

In order to refine our understanding and generate new hypothesis about the different biological systems involved in the pathogenesis of SCD we performed a meta-analysis of two recent gene expression studies involving patients with SCD. In addition, to explore the role of heme in the inflammatory response observed in these patients, we also performed meta-analyses comparing the gene expression pattern of heme-stimulated EC, with that observed in patients with SCD.

## Results

### Studies included in the meta-analysis

Four studies fulfilled the inclusion criteria and were selected for our meta-analysis. All of them provided high-quality metadata that allowed the meta-analysis. Table 1 provides the details of each study, and highlights the differences and similarities in sample type and microarray platform used. Two studies included samples from SCD patients (GSE53441 and GSE35007), and two studies included samples from EC stimulated with heme or with plasma from SCD patients. Samples from GSE35007 were further separated by us in two subgroups, according to disease status and to a severity score^15^, which were both informed in the database metadata. In total, 62 samples from patients with sickle cell anemia (homozygous SS) were included in the meta-analysis, of which 18 were in acute crisis and 44 were in steady-state. A subsample with the 56 samples with the top severity score (including patients in acute crisis and steady-state) was used in some of the analyses. Of note, all whole blood samples obtained from SCD patients were submitted to globin mRNA reduction, which according to a recent report, minimizes differences and increase the overlap with the gene expression signature of peripheral blood mononuclear cells (PBMC) in the context of SCD^16^.

### Biological terms identified in individual studies

We first identified relevant biological terms (pathways, transcription and kinase networks and gene ontology terms) enriched in each individual study that used samples from SCD patients (Table 2). The terms were identified using EnrichR, based on the list of DE up-regulated genes from each individual study. Pathways and gene ontology terms associated with immune response, autophagy, oxidative stress, heme and porphyrin metabolism, and coagulation were identified in this analysis, in both children and adults, steady-state and acute crisis (Table 2). Of note, these pathways were consistently identified in different gene set analysis libraries.

### Meta-analysis results of gene expression studies using samples from SCD patients

**Figure 1:**
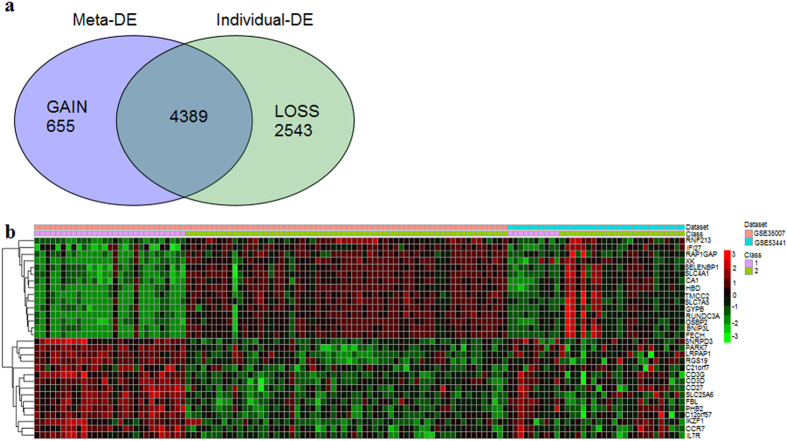
Gene expression pattern from the meta-analysis. The upper panel shows the overlap between DE genes identified in the meta-analysis (Meta-DE) and in each individual data analysis (individual-DE). Gain genes are those identified only in the meta-analysis. Loss genes are those identified in individual studies, but not in the meta-analysis. In the lower panel, a heatmap built using the top 30 differentially-expressed genes (15 up – and 15 down-regulated) comparing the gene expression pattern of studies that enrolled patients with sickle cell disease (GSE35007 and GSE53441) is shown. Class 1 and 2 refer to control and patient samples respectively, from each individual dataset.

We performed a meta-analysis of the two studies that evaluated gene expression signatures in patients with SCD (GSE53441 and GSE35007). Although the GSE35007 database includes 250 samples from children with SCD, we selected only SS homozygous patients with a severity score^15^ higher than 0.404 for our meta-analysis. In total, data from 62 patients were included, of which 44 were in steady-state and 18 in acute crisis. In total, 4,944 DE genes were identified, of which 655 were only identified in the meta-analysis (Fig. 1a). Using our more stringent criteria for differentially-expressed genes (>1.4 fold-change, up- or down-regulated in the same direction in both studies), a list of 384 DE genes (336 up-regulated and 48 down-regulated) was used in our subsequent analyses (supplementary table 1). A heatmap visualization of the gene expression pattern of the top 30 DE genes from the meta-analysis is presented in Fig. 1 (Fig. 1b). The top 10 up- and 10 down-regulated DE genes are presented in Table 3, along with the fold-change of expression in each individual study (Table 3).

To gain further insights into the biological processes associated with the expression signature identified in the meta-analysis, a comprehensive gene set analysis was performed, using EnrichR and the list of DE genes. The top significant (ranked by the *P-value*) biological pathways, gene ontology terms, protein-protein interaction (PPI) hub proteins and kinases predicted from the meta-analysis are shown in Table 4. Pathways associated with heme metabolism, innate immunity, proteasome degradation and autophagy were overrepresented in this analysis. In regard to PPI hub proteins and kinase enrichment analysis, the most significant predicted proteins were associated with ubiquitin-proteasome pathway, cell proliferation and motility, immune response and angiogenesis (Table 4). Again, all top-ranked pathways and terms were consistently identified in different gene set libraries.

**Figure 2:**
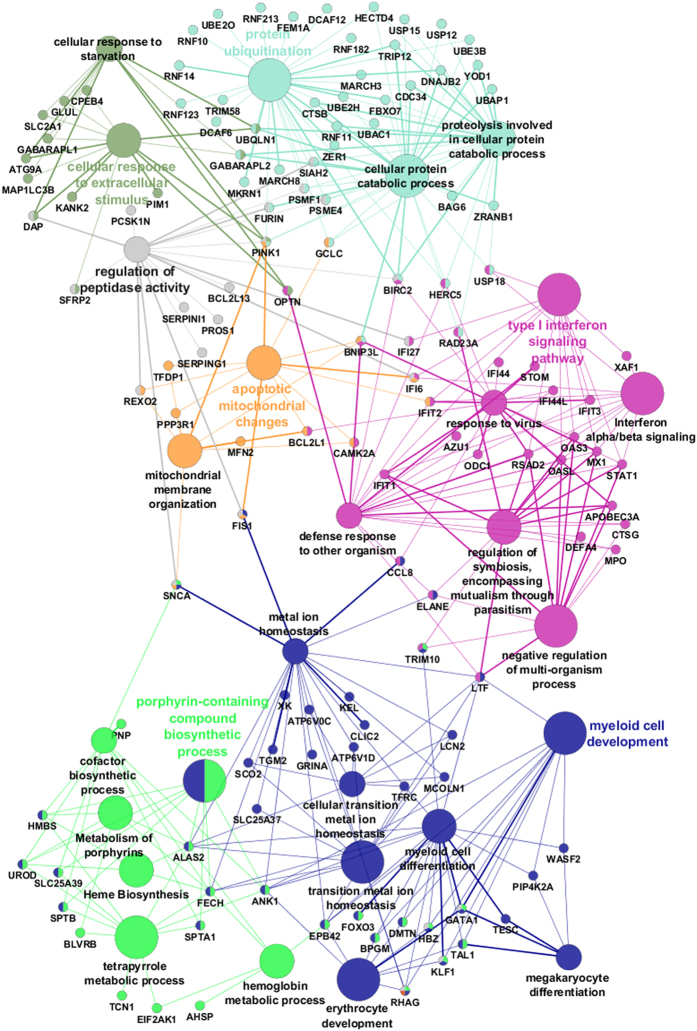
Enriched gene ontology pathways identified in the meta-analysis. The top enriched biological processes predicted from the list of up-regulated genes generated in the meta-analysis of samples from patients with sickle cell disease were grouped with the software ClueGO as a functional cluster (using a kappa score = 0.3). Each node represents a biological process. Their associated genes are represented as dots. Node and dot colors represent the functional group to which they belong. Mixed coloring nodes and dots belong to multiple groups. One ungrouped term is shown in grey. Edges represent term-term interaction or term-genes interaction. The title of the most significant term per group is shown in the network as a group title (colored text). The size of nodes reflects the enrichment significance of the terms.

Finally, to further facilitate the interpretation of the biological process associated with the gene signature from our meta-analysis, we used a bioinformatics tool that clusters gene ontology and pathway terms that participate in the same biological function, thereby reducing redundancy of these analyses. The tool also permits the visualization of gene interactions inside each cluster, as well as between different groups. The following groups were highly enriched: cellular response to extracellular stimulus, protein ubiquitination, type I interferon signaling pathway, porphyrin compound biosynthesis, myeloid cell development, apoptotic mitochondrial changes and regulation of peptidase activity. Figure 2 shows the relationship between these pathways, and the DE genes identified in our meta-analysis (Fig. 2).

### Meta-analysis results of gene expression studies with heme-stimulated endothelial cells

In order to study the mechanisms by which heme interferes with the pathogenesis of SCD, we next performed three exploratory meta-analysis between a gene expression study of heme-stimulated EC (GSE25014), and 3 different gene expression studies, respectively: (i) EC exposed to plasma from SCD patients during acute chest syndrome (GSE1849), (ii) SCD adult patients in steady-state (GSE53441), and (iii) SCD children in acute crisis (GSE35007). The list of top 10 DE genes is presented for each meta-analysis (Table 5). The gene coding for interferon alpha-inducible protein 27 (*IFI27*), which was up-regulated in the meta-analysis involving SCD patients, was again up-regulated in both meta-analyses involving heme-stimulated EC. In addition, the gene coding for Bone Marrow Stromal Cell Antigen-1 (*BST2*), which is involved in B cell growth and redox reactions was also identified in more than one meta-analysis (Table 5).

A gene set functional analysis was then performed to identify biological pathways and gene ontology terms overrepresented in each of the three meta-analyses using data from heme-stimulated EC. Again, pathways and gene ontology terms associated with coagulation and platelet activation, innate immune response, response to oxidative stress and angiogenesis were consistently overrepresented in these meta-analyses (Table 6).

### Prediction of the regulatory networks upstream to the DE genes identified in the meta-analysis

To gain insight into the regulatory system upstream of the DE genes identified in the meta-analysis, we used a bioinformatics tools (Expression2Kinase) to identify: (i) the predicted transcription factors (TFs) that likely drove the identified expression pattern; (ii) the intermediate proteins that could be forming a regulatory complex with these TFs; and (iii) the kinases most likely involved in the formation and activation of the regulatory complexes identified. With the candidate regulatory proteins identified, we built a subnetwork/protein complex that connects the TFs to each other and to their activation systems (Figure S1). The top 10 predicted TFs sorted by *p-value*, as well as the top 10 predicted kinases that might control the formation of these TF complexes are shown in supplementary Table 2 (Table S2).

## Discussion

Sickle cell disease is a monogenic, yet multisystem chronic progressive disease characterized by tissue damage and complications in nearly every body system^1^. This apparent paradox can be reconciled by considering SCD as a condition in which the function of almost every single gene is altered by the presence of hemoglobin S (HbS), as previously stated about cystic fibrosis^17^. Systemic and sustained inflammation, with the endothelium as the main target organ, is currently the best explanation for the multisystem nature of SCD. In this regard, several details on how the immune system ultimately responds to HbS polymerization - the initial trigger of the pathogenic cascade of SCD - have been described. This complex immune response involves endothelial and coagulation activation^18^,^19^, increased cellular adhesion^20^, expression of pro-inflammatory and pro-angiogenic cytokines^21^, neutrophil activation^22^ and increased oxidative stress^23^. Although critical to our understanding of SCD pathogenesis, these studies do not explain how exactly inflammation is elicited in the first place, nor the hierarchical relationship between all these elements. More recently, critical steps have been taken towards this goal, with the demonstration of the role of reperfusion injury^4^, nitric-oxide depletion by free hemoglobin^24^, and innate immunity activation by free heme^5^,^25^,^26^. Based on the analysis of this complex network of pathogenic mechanisms, and using a “systems biology” approach, Hebbel *et al.* proposed a probable hierarchy of sub-biological process involved in the pathogenesis of SCD, in which reperfusion injury was regarded as the most likely proximate mechanism of inflammation^4^. An updated version of this model by published by the same authors highlights the role of vascular stasis, free hemoglobin and free heme as initiators of inflammation^27^.

Here, we used a complementary strategy to further explore the relationship between the biological processes involved in the pathogenesis of SCD consisting of meta-analyses of public databases of gene expression studies. The past 15 years have witnessed an explosive increase in the number of studies using microarray technology in a variety of diseases and phenotypes^13^. With the adoption of reporting guidelines, and the establishment of public databases for raw microarray data, a large bulk of transcriptomic data from several diseases and phenotypes has accumulated, and is currently available for public reuse^11^. Frequently, the complexity of microarray data precludes the full exploration of its informative potential in original studies. In addition, inter-laboratory variation is still an inherent limitation of this technology^28^,^29^. By analyzing multiple experiments together, the effects of biases and artifacts can be reduced, helping true relationships to stand out^30^. Indeed, despite the heterogeneity of individual studies, meta-analysis of independent gene expression datasets were capable to identify new therapeutic targets in complex diseases^31^,^32^. This possibility has been further facilitated by the recent development of specific guidelines for these meta-analyses^12^,^13^, and of several bioinformatics tools capable to generate more complex and biologically relevant data from lists of DE genes^14^,^33^.

Robust experimental data demonstrate the prominent role of pathways associated with the innate immune system^34^,^35^, hemostasis^18^,^36^, and angiogenesis^21^ in the pathogenesis of SCD. The data generated by our analyses confirms the importance of these pathways in several ways. First, the top significant biological terms derived from the list of upregulated genes of each individual study included pathways associated with innate immunity (type I and II interferon signaling, defense response to virus, IL-6 signaling pathway), hemostasis (complement and coagulation cascades and MAPK signaling pathway), response to oxidative stress (glutathione metabolism, oxidative stress) and angiogenesis (adherens junction, MAPK signaling pathway). Second, the transcription signature derived from the meta-analysis of studies with SCD patient samples seemed to reflect a similar pattern, which was consistent across different gene set analysis libraries. Terms associated with innate immunity (interferon alpha/beta signaling, antiviral mechanisms by interferon-mediated genes, cellular response to type I interferon, response to other organisms and cytokine-mediated signaling pathway), heme biosynthesis (porphyrin and chlorophyll metabolism, metabolism of porphyrins), angiogenesis (signaling by TGF-beta receptor complex and degradation of extracellular matrix), autophagy, ubiquitination and proteasome degradation were also among the top ranked findings (Table 4). Third, among the top predicted hub proteins in protein-protein interactions and enriched kinases were proteins associated with innate immunity (TNFRSF1A, IRAK4), redox sensing (DYNLL1), autophagy (RPS27A, HSPA1A, UBC) and angiogenesis (SMAD4, BMPR2). Of note, mutations in the genes encoding BMPR2 and SMAD4 are among the most important genes associated with pulmonary hypertension^37^, a major clinical complication of SCD, and polymorphisms in *BMPR2* have been associated with an increased risk of this complication in SCD^38^. Moreover, the association between angiogenesis and pulmonary hypertension in SCD is also suggested by the demonstration of the prominent role of PlGF (Placental Growth Factor) in the pathogenesis of this condition^39^.

Despite the inherent limitation of analyzing lists of individual DE genes, the list generated by our meta-analysis (Table 3) also supports the role of these pathways in SCD. Interestingly, the list of up-regulated genes was higher in terms of absolute fold-change. The exact reason for this observation is not clear, but since recent bioinformatics studies demonstrated that a gene expression signature can be captured by the analysis of a subset of the most representative DE genes, we focused some of our analysis on these genes^40^. Nonetheless, the full list of DE genes (up and down-regulated) is presented in supplementary table S1, allowing additional analysis and hypothesis generation. Not surprisingly, 5 of the top 10 up-regulated genes were genes directly associated with erythrocyte development: hemoglobin delta, glycophorin B, carbonic anhydrase, Kell blood group precursor and Band 3 anion transport protein. These results probably reflect the contribution of transcripts from reticulocytes and nucleated red blood cells (NRBC) to gene expression studies involving patients with SCD and other conditions with high reticulocyte counts. In fact, it has been suggested that reticulocytes cannot be completely eliminated from PBMC prepations^41^. In addition, globin mRNA reduction doesn’t reduce other erythroid genes. To estimate the relative contribution of this erythroid development signature to our dataset, we compared our results with a dataset of the human reticulocyte transcriptome^42^. Of the 384 DE genes from our meta-analysis, 43 (11.2%) belonged to the list of the top 127 genes that are DE in human reticulocytes^42^ We also repeated the gene set analysis using EnrichR after the exclusion of these 43 genes, with similar results (data not shown). While corroborating the hypothesis that reticulocytes can copurify with PBMC, the results of this comparison suggest that the erythroid development signature is not cancelling out the representation of other genes in our dataset. Moreover, they suggest that the 5 additional upregulated genes which have not been previously associated with SCD, should be regarded as interesting targets for future studies. These genes include *RUNDC3A* (RUN Domain Containing 3A) and *RAP1GAP* (RAP1 GTPase Activating Protein), which are involved in the regulation of GTPase activity. RAP1GAP was also upregulated in monocytes from patients with chronic lymphocytic leukemia^43^, and RAP1GAP-mediated inhibition of RAP1 GTPase has been shown to severely impair macrophage function *in vitro*^44^; *TMCC2* (Transmembrane And Coiled-Coil Domain Family 2), a transmembrane protein whose functions are not well characterized, but that was recently associated with mean platelet volume in a large GWAS study^45^. *OSBP2*, which codes for Oxysterol binding protein 2 was also upregulated. Oxysterols are cytotoxic components that increase oxidative stress and have been shown to be increased in red cells from SCD patients^46^. More recently, the oxysterol 27-hydroxycholesterol has been shown to down-regulate heme-oxygenase and *NRF2*, a major anti-oxidant transcription factor, in astrocyte cells^47^. These results warrant further studies on the role of OSBP2 in SCD. Finally, *IFI27* (Interferon, Alpha-Inducible Protein 27) was upregulated in both meta-analyses, of clinical samples and heme-stimulated cells. In two recent publications, this protein was identified as a mediator of vascular injury in inflammatory animal models^48^,^49^. Of note, the gene coding for heme oxygenase 1 was up-regulated in the meta-analysis, but with a fold-change level lower than our 1.4 fold-change threshold for inclusion in the study. In regard to the list of downregulated genes, we would like to highlight the potential role of *PARK7*, a gene that encodes a redox-sensitive hypoxia-induced protein associated with cell death from acute ischemia-reperfusion injury^50^,^51^.

We also used the gene list generated in our meta-analysis to predict gene regulatory network associated with SCD, and generate new hypothesis about the regulatory complexes governing these gene expression patterns. CREB1 (*cAMP responsive element binding protein 1*) was identified as the most significant transcription factor. CREB1 regulates cell proliferation, survival and differentiation by mediating the transcription of genes containing a cAMP-responsive element. These include several genes from the immune system such as IL-2, IL-6, IL-10 and TNF-α. It has been proposed that phosphorylated CREB1 directly inhibits NF-κB activation, limiting pro-inflammatory responses^52^. Accordingly, several CREB1 targets were up-regulated in our meta-analysis such as FOXO3, NFE2, STAT1 and the kinase MAP2K3 (Table S1).

SCD patients present higher plasma concentration of heme which, according to recently generated data, can activate innate immunity in a TLR4-dependent fashion^25^, and act as an initiator of vaso-occlusion and ACS^5^,^26^. In addition, the endothelium has been increasingly recognized as a critical organ in the pathogenesis of both chronic and acute complications of SCD^53^. Accordingly, a secondary objective of our study was to identify genes and pathways that were stimulated by heme (in a controlled *in vitro* experiment using EC)^10^, and that were also DE in more clinically-oriented gene expression studies involving (1) EC stimulated by plasma from patients with ACS^54^ and (2 and 3) samples from patients with SCD^8^,^9^. One limitation of these 3 meta-analyses is that they compare gene expression patterns of different cells types (EC x whole blood/PBMC). Therefore, while DE genes and pathways that stand out were regarded by us as potential modulators of the effects of heme in the pathogenesis of SCD, no conclusion should be drawn about genes or pathways that were not identified. Nonetheless, considering the evidences supporting that free heme is a critical pathogenic mediator in SCD, and the limitations to study its effects in more relevant *in vivo* human models, we believe that our strategy could add relevant information about the role of heme in the pathogenesis of SCD. Interestingly, pathways and gene ontology terms identified in these 3 meta-analyses were similar to those identified in the meta-analysis between clinical samples, in that innate immunity, hemostasis, angiogenesis, response to oxidative stress and porphyrin metabolism dominated the top enriched terms (Table 6). In addition, the activation of these pathways is in accordance with recent data demonstrating the role of heme as a mediator of coagulation activation^55^ and PlGF-mediated pulmonary hypertension^56^. The list of up- and down-regulated genes reflects the enrichment of these pathways (Table 5).

Finally, using the meta-analysis data, we generated a network representing the most relevant and non-redundant biological processes associated with our results. As expected, this network captured the importance of erythroid/myeloid cell development and heme biosynthesis in SCD, as discussed above. Interestingly, it also highlighted the importance of innate immunity, response to oxidative stress, and ubiquitination, as the most important biological pathways associated with our results (Fig. 2). Innate immunity activation involves several pathways that were overrepresented in our study and that have been associated with SCD such as hemostasis and angiogenesis. These pathways can be viewed as parts of the body’s armamentarium for pathogen clearance and damage repair^57^. In recent years, the pathogenesis of SCD has been largely attributed to the detrimental role of erythroid DAMPs (danger associated molecular patterns) such as heme and free hemoglobin, as inflammatory drivers^58^. As a ubiquitous molecule in several domains of life, activation of innate immunity by heme has been regarded as a selected mechanism against invading pathogens, and hemorrhage-mediated tissue damage^25^. In this context, the constitutive activation of innate immunity in SCD could be viewed as a consequence of the evolutionary trade-off between the benefits of sensing heme and free hemoglobin as alarm signals, and the detrimental effects thereof, in the relatively rare individuals in whom high levels of circulating heme and free hemoglobin overcome the capacity of natural scavenging mechanisms. We believe that the prominent role of innate immunity and damage repair-associated pathways identified in our study further supports the model of erythroid DAMPs as critical triggers and perpetuators of inflammation in SCD. Our study has several limitations worth noting. First, meta-analysis of gene expression studies are a relatively recent strategy, with pros and cons. Although recent guidelines tried to establish minimum standards for these studies^12^, different statistical protocols can influence the results. We selected only published studies, with high-quality metadata, from internationally-recognized groups. The meta-analysis was performed with the INMEX tool, which uses one of the best statistical methods^59^ and has been used in several recent publications^60^,^61^,^62^. Second, the unbalanced contribution of reticulocytes transcripts to our results should be regarded as a potential bias, although the comparison between our meta-analysis and a published erythroid development signature suggests a limited influence of the latter on our results. A third limitation was the use of different sample types. In our main meta-analysis mRNA was obtained from whole blood and mononuclear cells respectively. Unfortunately, there were no databases available from studies with the same sample type. However, we chose to compare these two studies because whole blood samples from GSE53441 were submitted to a globin mRNA reduction step, which according to a well-designed study in sickle cell disease, minimizes differences and significantly increases the overlap of the gene expression profile compared to mononuclear cells^16^. Finally, we did not perform a qPCR validation step of our findings. It should be born in mind, however, that the objective of our study was not to revalidate these microarray data, but rather, to perform a global and hypothesis-generating transcriptomic analysis of SCD, that could be used to support independent investigations in the future.

In conclusion, our results demonstrate that the well-characterized association between innate immunity, hemostasis, angiogenesis and heme metabolism with SCD is consistently observed at the transcriptomic level across independent gene expression studies. We also generated a large database of candidate genes, pathways, transcription factors and kinases less or not previously associated with SCD that might be helpful for future studies about the pathogenesis of this complex disease.

## Methods

### Identification of eligible data sets

Microarray datasets that examined potentially DE genes in SCD, and that were publicly available by May 2015 were searched in two public repositories: NCBI Gene Expression Omnibus (GEO) (http://www.ncbi.nlm.nih.gov/geo/), and Array-express (http://www.ebi.ac.uk/arrayexpress/). Search was conducted with the terms (“sickle cell disease” and “homo sapiens”). Only studies that offered sufficient metadata for the analysis were included. GEO accession number, sample type, platform used for gene expression analysis, number of samples and gene expression data were extracted from the databases for each study.

### Meta-analysis of microarray datasets

Meta-analysis were performed using a web-based tool named INMEX (Integrative Meta-analysis of Expression data), designed to support and facilitate meta-analysis of multiple gene-expression data sets^63^. Gene expression data tables were constructed with downloaded raw data, following the INMEX recommendations^63^. All datasets were processed and annotated accordingly. Data integrity was checked for all datasets, and the meta-analysis was performed using the combined rank orders method, based on the RankProd package^59^,^63^. Briefly, fold changes (FC) between stimulated and control samples were computed for each dataset, and for all possible pairwise comparisons. The ranks of the ratios of each comparison were then used to calculate the rank product for each gene. Permutation tests were performed to assess the null distributions of the rank product within each data set. The whole process repeats multiple times to compute *p-value* and false discovery rate (FDR) associated with each gene^63^. Selected genes were ranked based on the combined rank product (combinedRP), which is a non-parametrical statistical method based on the rank of FC for each gene. The method is based on the assumption that if a gene appears repeatedly at the top of up- or down-regulated gene lists in replicate experiments, it is more likely a DE gene^59^. The lower the combinedRP value, the more interesting the candidate gene for differential expression^64^. DE genes identified in the meta-analysis were further selected using more stringent criteria, which was of a fold-change (FC) expression ≥1.4. We also excluded all genes identified in the meta-analysis that were DE in opposing directions. These post-processing steps were performed using a script in R language^65^. The GeneVenn web tool was used to examine the overlap of gene lists from our analysis^66^. A *p-value* < 0.05 was considered statistically significant. Heatmap visualization of a subset of genes from different studies was performed using the “Pattern extractor” tool from INMEX.

### Functional gene set analysis

In order to obtain additional biological information from the list of DE genes, a comprehensive functional gene set analysis was performed using the EnrichR platform^33^, a bioinformatics web-based tool that includes 69 gene-set libraries, such as KEGG, Wikipathways, as well as libraries that are only available in Enrichr. Libraries are divided into six categories: transcription, pathways, ontologies, diseases/drugs, cell types and miscellaneous, and the tool provides the possibility to obtain a comprehensive functional analysis using any gene list of interest. To further improve the interpretation of the biological significance of the enrichment terms obtained from Enrichr, we used the ClueGO^67^, a plug-in of Cytoscape^68^. This plug-in integrates the full list of identified gene ontology terms and pathways, and organizes them in functionally grouped networks, which depict the biological relationship between the pathways and gene ontologies. Briefly, we used two-sided (enrichment/depletion) hyper-geometric distribution tests, with a *p-value* significance level of ≤0.05), followed by the Bonferroni adjustment for the terms and the groups created by ClueGO. Fusion criteria to reduce the redundancy of the terms that have similar associated proteins was also applied allowing the maintenance of the most representative “parent” or “child” terms in the generated networks. The Kappa-statistics score threshold was set to 0.3, and leading term groups were selected based on the highest significance^69^.

### Regulatory gene network analysis

To further refine our exploratory study, we analyzed the regulatory networks of transcription factors and kinases predicted from the list of up-regulated genes derived from the meta-analysis, and from each individual gene expression study. Each gene list was uploaded into the transcription factor (TF) inference module of Expression2Kinases (X2K) software, and the TFs that were most likely involved with identified genes were extracted using the Position-Weight-Matrices (PWMs) database^70^. In addition, the top 10 list of human TFs ranked based on *p-value* was uploaded on Genes2Networks (G2N) module of X2K to identify transcriptional complexes associated with these gene signatures. These complexes were used to identify the protein kinases that are most likely responsible for TF complex formation and functional regulation. Finally, the regulatory network was visualized using yEd Graph Editor^71^.

## Additional Information

**How to cite this article**: Hounkpe, B. W. *et al.* Role of innate immunity-triggered pathways in the pathogenesis of Sickle Cell Disease: a meta-analysis of gene expression studies. *Sci. Rep.* **5**, 17822; doi: 10.1038/srep17822 (2015).

## Supplementary Material

Supplementary Information

## Figures and Tables

**Table 1:** Characteristics of individual studies included in the meta-analysis.

	Sample characteristics				Platform
	GEO accessionnumber	Size (Pt:Ctl)	Source	Experimental context	
1	GSE53441	24:10	PBMC	Adults, steady-state	Affymetrix Human U133 2.0 Plus
2	GSE35007*	62:29	Whole blood**	Children, acute crisis and steady-state	Illumina HumanHT-12 v4
4	GSE25014	12:12	PAEC/PMVEC	Heme-stimulated endothelial cells	Affymetrix Human U133 2.0 Plus
5	GSE1849	12:20	PAEC	Plasma-stimulated endothelial cells	Affymetrix Human Genome U133

GEO: Gene Expression Omnibus, Pt:Ctl: patients:controls; PBMC: peripheral blood mononuclear cells; PAEC: Human pulmonary artery endothelial cells, PMVEC: human pulmonary microvascular endothelial cells. *62 samples were selected samples from GSE35007, including 18 patients in acute crisis and 44 in steady-state (based on the highest severity-score informed in the database); **submitted to a globin mRNA reduction step.

**Table 2:** Significant biological terms identified in each individual study.

Steady state adults (GSE53441) – PBMC		
*Biological term*	*GSA library*	*P value*
B cell receptor signaling pathway	KEGG	0.0003
Type II interferon signaling	Wikipathways	9.2 e-7
Hemoglobins chaperone	Biocarta	6.0 e-6
Defense response to virus	GO biological process	3.9e-19
SYK (spleen tyrosine kinase)	KEA	8 e-5
ROCK2 (Rho-associated protein kinase 2)	Kinases perturbations	0.002
Top severity score - children (GSE35007) – whole blood		
MAPK signaling pathway	KEGG	0.02357
Adherens junction	KEGG	0.04524
Glutathione metabolism	Wikipathways	0.00091
Oxidative Stress	Wikipathways	0.00442
Heme biosynthesis	Wikipathways	0.00925
IL-6 signaling pathway	Wikipathways	0.0288
Autophagy	GO biological process	1.3 e-6
Regulation of cellular response to stress	GO biological process	0.00008
Acute crisis children (GSE35007) – whole blood		
Porphyrin and chlorophyll metabolism	KEGG	0.014
Complement and coagulation cascades	KEGG	0.016
Oxidative Stress	Wikipathways	5.6 e-4
Heme biosynthesis	Wikipathways	0.005
Response to virus	GO biological process	2.5 e-8
Autophagy	GO biological process	8.0 e-9
Response to type I interferon	GO biological process	1.0 e-8

GSA (gene set analysis) was performed using the EnrichR tool, that includes 69 different gene set libraries. A list of all significantly upregulated genes from each individual study was obtained using the GEO2R tool, from the Gene Expression Omnibus database. KEGG: Kyoto Encyclopedia of Genes and Genomes; GO: gene ontology term; KEA: kinase enrichment analysis; PBMC: peripheral blood mononuclear cells.

**Table 3:** Top 20 DE genes identified in the meta-analysis of studies with clinical samples.

	Fold-change in individual studies (LogFC)		Meta-analysis results		
**Genes**	GSE53441	GSE35007	CombRP	AveLogFC	P
*Up-regulated genes*					
*HBD*	3.80	4.12	6.37	3.96	<0.0001
*GYPB*	1.60	3.50	20.40	2.55	<0.0001
*CA1*	1.88	3.32	24.23	2.60	<0.0001
*RUNDC3A*	0.90	3.12	35.67	2.01	<0.0001
*RAP1GAP*	0.24	3.38	36.50	1.81	<0.0001
*IFI27*	2.70	3.07	37.90	2.88	<0.0001
*OSBP2*	0.73	3.05	40.60	1.88	<0.0001
*TMCC2*	1.16	2.99	40.62	2.07	<0.0001
*XK*	1.08	2.94	41.43	2.01	<0.0001
*SLC4A1*	2.44	2.82	42.16	2.63	<0.0001
*Down-regulated genes*					
C12orf57	−0.30	−1.24	156.78	−0.77	<0.0001
CD3G	−0.31	−1.23	176.91	−0.77	<0.0001
CCR7	−0.23	−1.17	206.27	−0.70	<0.0001
IL7R	−0.22	−1.15	231.82	−0.68	<0.0001
RGS19	−0.09	−1.09	267.28	−0.59	<0.0001
C21orf7	−0.11	−1.05	270.6	−0.58	<0.0001
SNRPD3	−0.09	−1.11	293.33	−0.60	<0.0001
LRPAP1	−0.05	−1.04	306.19	−0.54	<0.0001
PARK7	−0.07	−1.05	309.77	−0.56	<0.0001
PHB2	−0.20	−0.99	333.57	−0.59	<0.0001

Genes were ranked based according to the combined-rank product obtained in each meta-analysis. LogFC: base 2 log of Fold-change; CbnRP: combined Rank Product (the smaller the combRP, the higher is the likelihood of differential expression; AveLogFC: average LogFC.

**Table 4:** Top biological pathways and terms identified by GSA in the meta-analysis of studies with clinical samples.

Biological pathway	Overlap	GSA library	*p-value*
Porphyrin and chlorophyll metabolism	05/41	KEGG	9.0 e-4
Interferon alpha/beta signaling	12/67	Reactome	1.7 e-8
Metabolism of porphyrins	05/17	Reactome	0.007
Antigen processing: ubiquitination & proteasome degradation	10/211	Reactome	0.007
Antiviral mechanism by IFN-stimulated genes	05/71	Reactome	0.01
Apoptosis	07/146	Reactome	0.02
Class I MHC mediated antigen processing & presentation	10/255	Reactome	0.025
Degradation of the extracellular matrix	05/89	Reactome	0.03
Cell surface interactions at the vascular wall	05/99	Reactome	0.043
Signaling by TGF-beta receptor complex	04/70	Reactome	0.048
Heme biosynthesis	04/09	Wikipathway	4.7 e-5
Senescence and autophagy	6/108	Wikipathway	0.012
**Gene ontology term**	**Overlap**	**GSA library**	***p-value***
Tetrapyrrole metabolic process	11/59	GO	3.2 e-8
Cellular response to type I interferon	11/65	GO	7.9 e-8
Response to other organism	26/462	GO	4.5 e-7
Autophagy	12/102	GO	7.2 e-7
Cytokine-mediated signaling pathway	15/342	GO	1.6 e-3
**PPI hub proteins**	**Overlap**	**GSA library**	***p-value***
RPS27A	11/173	EnrichR	3.0 e-6
HSPA1A	9/145	EnrichR	3.0 e-5
DYNLL1	9/183	EnrichR	1.9 e-4
TNFRSF1A	8/173	EnrichR	6.0 e-4
SMAD4	9/221	EnrichR	7 e-4
UBC	16/540	EnrichR	2.5 e-4
**Kinase enrichment analysis**	**Overlap**	**GSA library**	***p-value***
BMPR2	194/10324	EnrichR	0
IRAK4	70/2805	EnrichR	4.7 e-9

GSA (gene set analysis) was performed using the EnrichR tool, that includes 69 different gene set libraries. Genes or terms were ranked based on the *p-value*. Overlap indicates the number of hits from the meta-analysis compared to each curated gene set library. GO: gene ontology biological process.

**Table 5:** Top 10 DE genes identified in the meta-analysis between heme-stimulated endothelial cells and clinical samples.

Heme-stimulated endothelial cells x plasma(ACS)-stimulated EC					
*Up-regulated*			*Down-regulated*		
Gene name	AveLogFC	*p-value*	Gene name	AveLogFC	*p-value*
*LYPD1*	4.10	<0.0001	*FGF13*	−4.12	<0.0001
*BST2*	4.20	<0.0001	*PDPN*	−4.22	<0.0001
*DARC*	3.82	<0.0001	*PLA1A*	−3.67	<0.0001
*C7*	3.77	<0.0001	*PGM5*	−3.90	<0.0001
*CCL23*	3.72	<0.0001	*MRC1*	−3.80	<0.0001
Heme-stimulated endothelial cells x SCD adults (PBMC/steady state)					
*IFI27*	4.65	<0.0001	*CA2*	−4.40	<0.0001
*IFI44L*	3.90	<0.0001	*KBTBD11*	−3.00	<0.0001
*SELENBP1*	3.00	<0.0001	*KRT23*	−2.16	<0.0001
*IFIT1*	2.01	<0.0001	*HSD17B2*	−4.36	<0.0001
*BST2*	4.26	<0.0001	*GPAT2*	−2.36	<0.0001
Heme-stimulated endothelial cells x SCD children (whole blood/acute crisis)					
*IFI27*	4.85	<0.0001	*TCL1A*	−0.98	<0.0001
*CA1*	1.60	<0.0001	*CLC*	−0.80	<0.0001
*TMCC2*	1.42	<0.0001	*C12orf57*	−1.14	<0.0001
*SELENBP1*	3.34	<0.0001	*CD3G*	−0.84	<0.0001
*RUNDC3A*	1.44	<0.0001	*ITM2C*	−2.75	<0.0001

In the first panel, we present the results of the meta-analysis between the study comparing heme-stimulated endothelial cells with plasma (from patients with ACS)-stimulated endothelial cells. In the next two panels, we present the results of the meta-analysis between heme-stimulated endothelial cells versus clinical samples. Genes were ranked based according to the combined-rank product obtained in each meta-analysis. ACS: acute chest syndrome; AveLogFC: average (from both studies) of the base 2 log of Fold-change; PBMC: peripheral blood mononuclear cells.

**Table 6:** Top biological pathways and terms identified by gene set analysis in the meta-analysis between heme-stimulated endothelial cells versus other gene expression studies in sickle cell disease.

Biological pathway	Library	Meta-analysis
Complement and coagulation cascades	KEGG	1, 2, 3
Cytokine cytokine receptor interaction		1, 2
Porphyrin and chlorophyll metabolism		3
Glutathione metabolism		3
Cell adhesion molecules		1
Interferon alpha/beta signaling	Reactome	1, 2, 3
Extracellular matrix organization		1, 2, 3
Cell surface interactions at the vascular wall		1
Synthesis of prostaglandins and thromboxanes		1, 3
Platelet degranulation		1, 3
VEGF binds to VEGFR		1, 3
Formation of Fibrin Clot		1
Heme Biosynthesis	Wiki	3
Oxidative Stress		2, 3
Transcriptional activation by NRF2	Pathways	2
Cytokines and inflammatory response		1
**Gene ontology term**	**Library**	**Meta-analysis**
Defense response to other organism	Gene ontology biological process	1, 3
Cellular response to type I interferon		1, 2, 3
Inflammatory response		1, 3
Regulation of angiogenesis		1,2, 3
Regulation of vasculature development		1, 3
Cellular response to cytokine stimulus		1, 3
Blood coagulation		1, 2
Platelet activation		1, 3
Regulation of cellular response to VEGF stimulus		1, 3
Regulation of vascular permeability		2
Regulation of cell adhesion		2
Positive regulation of vasodilation		3
Response to hypoxia		3
Cellular response to reactive oxygen species		3
Regulation of acute inflammatory response		3
NIK/NF-kappaB signaling		3

GSA (gene set analysis) was performed using the EnrichR tool, that includes 69 different gene set libraries. Pathways or terms identified in the meta-analysis between heme-stimulated endothelial cells versus 3 additional gene expression studies involving SCD patient samples are presented. The meta-analysis compared heme-stimulated endothelial cells versus: (1) adults in steady-state; (2) pulmonary endothelial cells stimulated with plasma from patients with SCD during acute chest syndrome; and (3) children with SCD in acute crisis.
