# Supplementary material for: Role of innate immunity-triggered pathways in the pathogenesis of Sickle Cell Disease: a meta-analysis of gene expression studies
Source: Sci Rep. 2015 Dec 9;5:17822. doi: 10.1038/srep17822 (PMC4673434; doi:10.1038/srep17822)
Supplement: Supplementary Information [file srep17822-s1.doc]

**Supplementary information**

**Role of innate immunity-triggered pathways in the pathogenesis of Sickle Cell Disease: a meta-analysis of gene expression studies**

Authors: Bidossessi Wilfried Hounkpe ^1^, Maiara Marx Luz Fiusa ^1^, Marina Pereira Colella ^2^, Loredana Nilkenes ^1^, Rafaela Benatti de Oliveira ^1^, Sara T Olalla Saad ^1^, Fernando Costa ^1^, Magnun Nueldo Santos ^1^, Erich Vinicius De Paula ^1^

Affiliations: ^1^ Faculty of Medical Sciences, University of Campinas, Campinas, SP, Brazil; ^2^ Hematology and Hemotherapy Center, University of Campinas, Campinas, SP, Brazil

Supplementary Table S1. Complete list of differentially-expressed genes obtained from the meta-analysis of gene expression studies with samples from SCD adult PBMC and SCD children whole blood (acute crisis).

| **Up-regulated genes** | | | | | | | | | | | | |
| --- | --- | --- | --- | --- | --- | --- | --- | --- | --- | --- | --- | --- |
| *ABCB10* | *CA1* | | | *EPB41* | *HBQ1* | *MFN2* | | | *PPP2R5B* | *SLC14A1* | *TRAK2* | |
| *ABCC13* | *CALCOCO1* | | | *EPB42* | *HBZ* | *MICAL2* | | | *PPP3R1* | *SLC1A5* | *TRIM10* | |
| *ABCC4* | *CALCOCO1* | | | *EPB49* | *HDGF* | *MICALCL* | | | *PROS1* | *SLC22A4* | *TRIM58* | |
| *ADIPOR1* | *CAMK2A* | | | *EPSTI1* | *HEMGN* | *MKRN1* | | | *PSME4* | *SLC25A37* | *TRIP12* | |
| *ADRA2C* | *CCL8* | | | *ESPN* | *HEPACAM2* | *MLPH* | | | *PSMF1* | *SLC25A39* | *TSPAN5* | |
| *ADRA2C* | *CCNDBP1* | | | *FAM100A* | *HERC5* | *MPO* | | | *R3HDM4* | *SLC2A1* | *TSPAN7* | |
| *AHSP* | *CDC34* | | | *FAM104A* | *HIST1H1C* | *MPP1* | | | *R3HDM4* | *SLC38A5* | *TSPO2* | |
| *AIDA* | *CEACAM8* | | | *FAM117A* | *HMBS* | *MX1* | | | *RAB2B* | *SLC4A1* | *TSTA3* | |
| *ALAS2* | *CHD8* | | | *FAM210B* | *HPS1* | *MXI1* | | | *RAB3IL1* | *SLC6A8* | *TTC25* | |
| *ALDH5A1* | *CHD8* | | | *FAM210B* | *IFI27* | *MYL4* | | | *RAD23A* | *SLC7A5* | *TTC25* | |
| *ANK1* | *CHPT1* | | | *FAM214B* | *IFI44* | *NAPA* | | | *RALBP1* | *SMOX* | *TTC7B* | |
| *ANKRD22* | *CISD2* | | | *FAM46C* | *IFI44L* | *NCOA4* | | | *RANBP10* | *SNCA* | *TUBB2A* | |
| *ANKRD22* | *CLIC2* | | | *FBXO34* | *IFI6* | *NFE2* | | | *RAP1GAP* | *SPTA1* | *UBAC1* | |
| *ANKRD9* | *CMAS* | | | *FBXO7* | *IFIT1* | *NFIX* | | | *RBM38* | *SPTB* | *UBAP1* | |
| *APOBEC3A* | *CMBL* | | | *FECH* | *IFIT2* | *NPL* | | | *REXO2* | *SRRD* | *UBE2H* | |
| *ARG1* | *CMBL* | | | *FEM1A* | *IFIT3* | *NPRL3* | | | *RHAG* | *SRSF8* | *UBE2O* | |
| *ARHGEF12* | *CMPK2* | | | *FHDC1* | *IGF2BP2* | *NSUN3* | | | *RHCE* | *SRXN1* | *UBE3B* | |
| *ARL4A* | *CNPPD1* | | | *FIS1* | *ISCA1* | *OAS3* | | | *RHD* | *ST13* | *UBE3B* | |
| *ASCC2* | *CPEB4* | | | *FKBP8* | *ITLN1* | *OASL* | | | *RIOK3* | *ST6GALNAC4* | *UBQLN1* | |
| *ATG9A* | *CREG1* | | | *FNBP1L* | *JAZF1* | *OASL* | | | *RMND5A* | *STAT1* | *UBXN6* | |
| *ATP6V0C* | *CSDA* | | | *FOXO3* | *KANK2* | *ODC1* | | | *RNF10* | *STOM* | *UROD* | |
| *ATP6V1D* | *CTNNAL1* | | | *FOXO4* | *KAT2B* | *OPTN* | | | *RNF11* | *STRADB* | *USP12* | |
| *AZU1* | *CTSB* | | | *FRMD4A* | *KEL* | *OR2W3* | | | *RNF123* | *TAL1* | *USP15* | |
| *BAG6* | *CTSG* | | | *FURIN* | *KIAA0430* | *OSBP2* | | | *RNF14* | *TBC1D10B* | *USP18* | |
| *BCL2L1* | *CYSTM1* | | | *GABARAPL1* | *KLC3* | *PARP9* | | | *RNF182* | *TBC1D22B* | *VWCE* | |
| *BCL2L13* | *DAP* | | | *GABARAPL2* | *KLF1* | *PCGF5* | | | *RNF213* | *TBCEL* | *VWCE* | |
| *BIRC2* | *DCAF12* | | | *GATA1* | *KRT1* | *PCSK1N* | | | *RPIA* | *TCN1* | *WASF2* | |
| *BLVRB* | *DCAF6* | | | *GCLC* | *LAMP3* | *PCTP* | | | *RSAD2* | *TCP11L2* | *WBP2* | |
| *BMP2K* | *DCAF6* | | | *GLRX5* | *LCN2* | *PGM2L1* | | | *RSRC1* | *TERF2IP* | *WDR26* | |
| *BNIP3L* | *DCUN1D1* | | | *GLUL* | *LGALS3* | *PHOSPHO1* | | | *RUNDC3A* | *TESC* | *WNK1* | |
| *BPGM* | *DEFA4* | | | *GMPR* | *LHFPL2* | *PIGQ* | | | *SAMD9* | *TFDP1* | *XAF1* | |
| *BSG* | *DNAJA4* | | | *GPR146* | *LPIN2* | *PIGQ* | | | *SAMD9L* | *TFDP2* | *XK* | |
| *C12orf51* | *DNAJB2* | | | *GRINA* | *LRRC8A* | *PIM1* | | | *SCO2* | *TFRC* | *XPO7* | |
| *C14orf45* | *DPCD* | | | *GSPT1* | *LTF* | *PINK1* | | | *SEC14L1* | *TGM2* | *YIPF6* | |
| *C22orf13* | *DPM2* | | | *GYPA* | *MAP1LC3B* | *PITHD1* | | | *SEC62* | *TMCC2* | *YOD1* | |
| *C22orf25* | *DPYSL5* | | | *GYPB* | *MAP2K3* | *PLEK2* | | | *SELENBP1* | *TMEM111* | *YPEL3* | |
| *C5orf4* | *DYSF* | | | *GYPE* | *MARCH3* | *PLVAP* | | | *SERPING1* | *TMEM158* | *YPEL4* | |
| *C6orf192* | *E2F2* | | | *HAGH* | *MARCH8* | *PNP* | | | *SERPINI1* | *TMEM56* | *YY1AP1* | |
| *C7orf41* | *EIF1B* | | | *HBBP1* | *MBNL3* | *PNPLA2* | | | *SESN3* | *TMEM63B* | *ZER1* | |
| *C7orf41* | *EIF2AK1* | | | *HBD* | *MBOAT2* | *POC1B* | | | *SFRP2* | *TMEM86B* | *ZMAT2* | |
| *C9orf40* | *ELANE* | | | *HBE1* | *MCOLN1* | *POLL* | | | *SIAH2* | *TMOD1* | *ZNF23* | |
| *C9orf78* | *ELOF1* | | | *HBM* | *MED25* | *PPME1* | | | *SIGLEC1* | *TNS1* | *ZRANB1* | |
| **Down-regulated genes** | | | | | | | | | | | | |
| *ATP6V0E2* | | *CD3D* | *CMTM8* | | *FBL* | | *LIME1* | *PARK7* | | *RPL35* | | *SIGLEC10* |
| *C12orf57* | | *CD3G* | *CRIP1* | | *GZMM* | | *LRPAP1* | *PHB2* | | *RPL4* | | *SLC25A6* |
| *C21orf7* | | *CD52* | *DDIT4* | | *IKZF1* | | *MIF* | *PMM1* | | *RPS23* | | *SNRPD3* |
| *CCR7* | | *CD7* | *DNLZ* | | *IL32* | | *MRPL40* | *RGS19* | | *RPS5* | | *THEMIS* |
| *CD2* | | *CD8A* | *EEF1D* | | *IL7R* | | *NELL2* | *RPL10A* | | *SF3A3* | | *TOB1* |
| *CD27* | | *CD96* | *EIF3L* | | *LEF1* | | *NOSIP* | *RPL23* | | *SGK223* | | *TXLNG2P* |

Supplementary Table S2. Top 10 transcription factors and kinases predicted by the meta-analysis of gene expression studies with samples from SCD adult PBMC and SCD children whole blood (acute crisis).

| **Transcription factors** | **Overlap** | ***p-value*** |
| --- | --- | --- |
| CREB1 | 65/2749 | 3.99 e-5 |
| PURA | 17/492 | 0.001 |
| WT1 | 58/492 | 0.001 |
| FOXJ1 | 36/2689 | 0.001 |
| NR1H3 | 34/1385 | 0.001 |
| CEBPD | 35/1495 | 0.003 |
| ATF4 | 32/1398 | 0.006 |
| ELK1 | 34/1511 | 0.007 |
| PLAU | 31/1362 | 0.008 |
| UBTF | 33/1493 | 0.01 |
| **Kinases** | | |
| MAPK1 | 44/229 | 6.3 e-15 |
| MAPK3 | 33/170 | 2.1 e-11 |
| TAF1 | 16/42 | 1.7 e-9 |
| CSNK2A1 | 31/194 | 6.6 e-9 |
| RPS6KA1 | 16/53 | 2.7 e-8 |
| RPS6KA4 | 9/10 | 3.3 e-8 |
| MAPK14 | 43/377 | 6.03 e-8 |
| RPS6KA2 | 10/21 | 4.43 e-7 |
| GSK3B | 49/501 | 4.97 e-7 |
| CDK2 | 42/398 | 6.53 e-7 |

Transcription factors and kinases were identified using the Expression2Kinase software, using the results of the meta-analysis between studies with samples from patients with SCD (adults; GSE53441 and children; GSE35007).


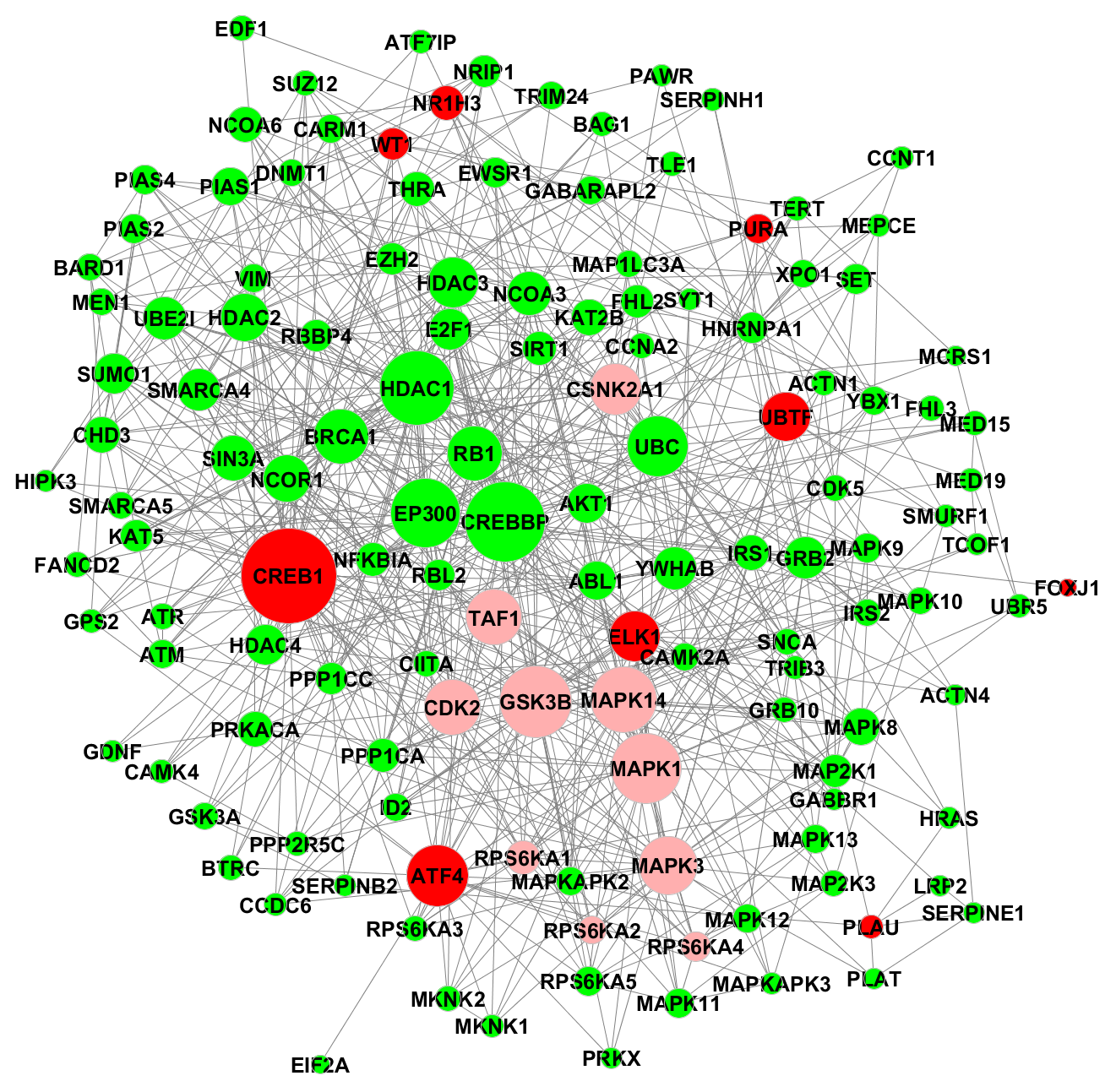
Figure S1. Transcriptional regulatory network based on the meta-analysis.

Regulatory network analysis was performed to determine the regulation complexes upstream of differentially expressed genes identified in the meta-analysis (predicted from the list of up-regulated genes). Red dots represent the transcription factors; pink dots represent the kinases; green dots represent the intermediate proteins of regulatory complexes. Node size reflects the importance of the protein in the network, according to the p-value: the higher the node, the more significant the protein in the network. Edges reflect the protein-protein interaction. The length of edge indicates the degree of relationship.
